# Clusters of comorbidities in fibrotic hypersensitivity pneumonitis

**DOI:** 10.1186/s12931-022-02291-4

**Published:** 2022-12-21

**Authors:** Thomas Skovhus Prior, Julia Wälscher, Benjamin Gross, Elisabeth Bendstrup, Michael Kreuter

**Affiliations:** 1 Centre for Rare Lung Diseases, Department of Respiratory Diseases and Allergy, Aarhus University Hospital, Aarhus, Denmark; 2 Department of Clinical Medicine, Aarhus University, Aarhus, Denmark; 3 Centre for Interstitial and Rare Lung Diseases, Pneumology and Respiratory Critical Care Medicine, Thoraxklinik, University of Heidelberg, Heidelberg, Germany; 4 German Center for Lung Research (DZL), Heidelberg, Germany; 5 Centre for Interstitial and Rare Lung Diseases, Pneumology Department, Ruhrlandklinik, University Hospital, University of Essen, Essen, Germany

**Keywords:** Comorbidity, Hypersensitivity pneumonitis, Allergic alveolitis, Cluster analyses, Interstitial lung disease, Pulmonary fibrosis, Respiratory tract diseases

## Abstract

**Background:**

Hypersensitivity pneumonitis (HP) is a type of interstitial lung disease (ILD) with a variable disease course and prognosis ranging from inflammatory and self-limiting to irreversible and progressive pulmonary fibrosis. Comorbidities are common in HP and may have an impact on prognosis. Due to the heterogeneity of HP presentation and progression, the identification of specific phenotypes in relationship to disease course and outcome is essential. The aim of this study was to identify clusters of comorbidities which could represent phenotypes in fibrotic HP and examine their impact on prognosis.

**Methods:**

Patients diagnosed with fibrotic HP at a tertiary referral center for ILD were included. Comorbidities were systematically registered and clusters of comorbidities were identified using cluster analyses. Disease progression and survival was estimated for each cluster.

**Results:**

The cohort comprised 211 patients with 53.6% males, mean age 63.0, baseline FVC 72.7%, DLCO 44.1%. Median follow-up time was 1.8 years (IQR 0.7–3.9). Three clusters with distinct comorbidity profiles and clinical characteristics were identified. One cluster dominated by elder male patients with predominantly cardiovascular diseases was associated with more respiratory hospitalizations and a worse prognosis. Differences in pulmonary function or exercise capacity trajectories between clusters were not observed.

**Conclusions:**

Three clusters with distinct comorbidities were identified and could represent phenotypes in fibrotic HP not previously recognized. The worst prognosis was observed in a cluster dominated by elder males with cardiovascular diseases. Increased focus on prevention and treatment of comorbidities could potentially improve the prognosis of patients with fibrotic HP.

## Background

Hypersensitivity pneumonitis (HP) is a subtype of interstitial lung disease (ILD) that can be inflammatory and/or fibrotic of nature. HP is typically caused by the inhalation of an overt or occult antigen resulting in an immune-mediated reaction affecting the lung parenchyma and small airways in susceptible individuals [1]. Multiple studies have shown that the disease course, treatment response, and prognosis is determined by the identification and eradication of the eliciting antigen, and by the presence of fibrosis [2–6]. Non-fibrotic HP may be self-limiting upon removal of the offensive antigen and has a better prognosis compared to fibrotic HP (fHP), where some patients will show an irreversible progressive fibrosing phenotype similar to idiopathic pulmonary fibrosis (IPF) [2]. Risk factors for a progressive fibrosing phenotype are not well defined but include continued exposure to the eliciting antigen, extent of fibrosis, and the type of fibrosis e.g., presence of honeycombing on high resolution computed tomography (HRCT) and in histopathology is associated to a worse prognosis [3–5]. The incidence of HP has been shown to increase especially in patients older than 55 years [3]. Due to the heterogeneity of HP presentation and progression, it could be valuable to identify if specific phenotypes are related to disease course and outcome.

Comorbidities are presumably common in most fibrotic ILDs [7], but have been best studied in IPF, where the number and specific type of comorbidities are associated with a worse outcome and health-related quality of life [8–10]. Similarly in HP, the burden of comorbidities is high and comorbidities are associated to mortality [11]. In a recent study, the most common comorbidities were arterial hypertension, gastro-esophageal reflux disease, diabetes, and coronary heart disease. However, there was no association between the absolute number of comorbidities and mortality. Pulmonary hypertension, diastolic dysfunction, and cerebrovascular disease were among the comorbidities most commonly encountered in non-survivors [11]. Cluster analysis has become an interesting strategy to determine individual groups or clusters of patients with homogenous presentation with respect to clinical characteristics, comorbidities, and prognosis [12]. Previous analysis in IPF identified four specific clusters of comorbidities that may represent specific phenotypes in IPF with different outcomes [8]. It is not known if specific combinations or clusters of comorbidities can help predict disease outcome in fHP and therefore, the aim of the present study was to identify clusters of comorbidities in patients with fHP and examine their prognosis.

## Methods

### Study subjects

Patients diagnosed with fHP between June 1995 and November 2017 at the tertiary referral center for ILD, Heidelberg, Germany were included in the study. All clinical diagnoses were based on multidisciplinary team discussions including pulmonologists, radiologists and pathologists experienced in ILD. HRCT scans were available for all patients, and histopathological samples were available for most patients (79%). The diagnostic process and associations between individual comorbidities and survival has been described in a previous paper [11].

### Study measures

Data regarding comorbidities, age, gender, smoking history, pulmonary function tests (forced expiratory volume in 1 s (FEV1), forced vital capacity (FVC), FVC/FEV1 ratio, total lung capacity (TLC), and diffusing capacity of the lung for carbon monoxide (DLCO)), long-term oxygen therapy (LTOT), 6-min walk test distance (6MWD), lymphocyte count in bronchoalveolar lavage (BAL), and respiratory hospitalizations were extracted from the database.

Registration of comorbidities was based on patient interviews, a standardized questionnaire for ILD, medical records and current medications[13]. The following comorbidities were assessed: airway obstruction, pulmonary hypertension, obstructive sleep apnea, arterial hypertension, ischemic heart disease, heart failure, heart valve disease, atrial fibrillation, other arrythmias, cerebrovascular disease, vascular disease, thromboembolic disorders, peripheral artery disease, chronic kidney disease, diabetes mellitus, osteoporosis, hypothyroidism, obesity, lung cancer, other cancers, anemia, liver disease, gastro-esophageal reflux disease, depression and anxiety.

### Statistical analyses

Discrete variables are presented as frequencies, and continuous variables are presented as median with interquartile range (IQR) or mean with standard deviation (SD).

Clusters of comorbidities were determined by self-organizing maps, also known as Kohonen maps, using Viscovery SOMine 7.2 (Viscovery Software GmbH, Vienna, Austria). This technique uses non-parametric regression analyses to transform multidimensional data into lower dimensional reflections. Data were analyzed for similarity using the SOM-Ward Cluster algorithm, and homogenous groups were visualized in attribute maps [11]. In these maps, the average frequency of each comorbidity was indicated by a fitted color scale. For comparison of each cluster against the two other clusters in combination, continuous data with a normal distribution were analyzed by the two-sided t-test with 95% confidence and otherwise by the Wilcoxon Mann–Whitney U test. Binary data were compared using the chi-squared test or Fisher’s exact test as appropriate.

The gender, age, physiology index for ILD (ILD-GAP) was calculated based on gender, age, and pulmonary function and adjusted for HP [14].

Kaplan–Meier curves, log-rank test, and univariate and multivariate Cox regression were used for mortality analyses based on all-cause mortality. Specific cause of mortality was compared between the clusters using Fisher’s exact test. Cox regression analyses were adjusted for GAP-ILD index and pack years. Changes in FVC% and DLCO% predicted during follow-up in the three comorbidity clusters were estimated by linear mixed effects models. Data were analyzed using STATA 14.2 (StataCorp, College Station, Texas).

## Results

### Characteristics of the comorbidity clusters

The study population comprised 211 patients with fHP (Table 1). The cohort had a slight majority of male patients (53.6%). The mean (SD) age at diagnosis was 63.0 (13.3) years. FVC and DLCO were reduced. Half of the cohort consisted of never smokers. Median (IQR) follow-up time was 1.8 (0.7–3.9) years.

**Table 1:** Baseline characteristics of the entire cohort and the three clusters

Clinical characteristics	Entire cohort (*n* = 211)	Cluster 1 (*n* = 99)		Cluster 2 (*n* = 65)		Cluster 3 (n = 47)
Male, *n* (%)	113 (53.6%)	57 (57.6%)		46 (70.8%) **		10 (21.3%) **
Age at diagnosis, years (SD)	63.0 (13.3)	59.3 (14.3) **		69.8 (10.2) **		61.3 (11.4)
Smoking status, *n* (%)						
Never	106 (50.2%)	47 (48.0%)		32 (49.2%)		27 (57.4%)
Former	95 (45.2%)	45 (45.9%)		32 (49.2%)		18 (38.3%)
Current	9 (4.3%)	6 (6.1%)		1 (1.5%)		2 (4.3%)
Pack years (IQR)	0 (0–20)	2 (0–20)		0 (0–30)		0 (0–10)
FVC, % predicted (SD)	72.7 (21.2)	72.9 (18.2)		74.0 (25.8)		70.3 (20.5)
TLC, % predicted (SD)	74.1 (15.9)	76.7 (14.6) *		71.3 (17.7)		72.6 (15.4)
DLCO, % predicted (SD)	44.1 (13.9)	46.3 (13.4)*		42.6 (14.9)		41.2 (13.2)
6MWD, m (SD)	373.4 (112.1)	402.0 (102.4)**		354.8 (109.0)		334.8 (122.8)*
Long-term oxygen therapy, *n* (%)	39 (18.5%)	12 (12.1%)*		18 (27.7%)*		9 (19.1%)
Lymphocytes in BAL, % of total (IQR)	24% (10–56%)	30% (14–61%)*		23% (6–48%)		21% (8–57%)
Biopsy (transbronchial cryobiopsi or surgical lung biopsy), *n* (%)	83 (39.3%)	45 (45.5%)		17 (26.2%)*		21 (44.7%)
ILD-GAP index, *n* (%)						
0–1	96 (45.5%)	57 (57.6%)	*	15 (23.4%)	**	24 (54.5%)
2–3	87 (41.2%)	34 (34.3%)		37 (57.8%)		16 (36.4%)
4–5	19 (9.0%)	6 (6.1%)		9 (14.1%)		4 (9.1%)
> 5	5 (2.4%)	2 (2.0%)		3 (4.7%)		0 (0.0%)

Values are presented as *n* (%), mean with standard deviation (SD) or median with interquartile range (IQR) [11]. Results from one cluster differing significantly from the two other clusters in combination are marked with: *p < 0.05; **p < 0.01

*FVC* forced vital capacity, *TLC* total lung capacity, *DLCO* diffusing capacity of the lung for carbon monoxide, *6MWD* 6-min walk test distance, *ILD* Interstitial lung disease, *GAP* gender, age and physiology index, *BAL* bronchoalveolar lavage, *HRCT* high-resolution computed tomography

**Fig. 1:**
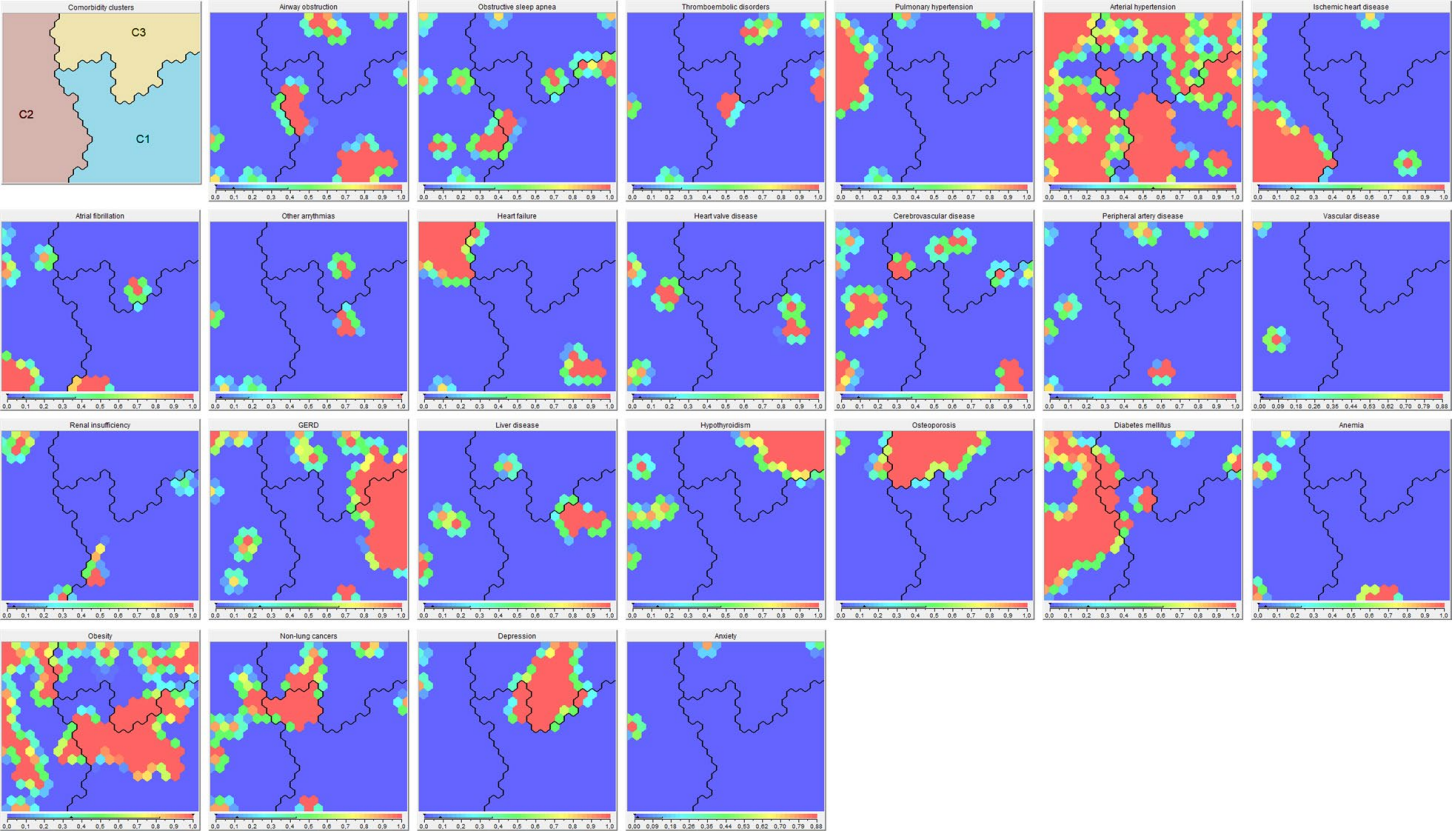
Attribute self-organizing maps for each comorbidity and clusters borders

Three clusters with distinct comorbidity profiles were identified (Tables 1, 2, Fig. 1). Patients in the first cluster were younger, had slightly higher TLC and DLCO and longer 6MWD. Fewer patients received LTOT, and lymphocyte count in BAL was higher compared to the two other clusters in combination. Patients in cluster 1 had fewer total number of comorbidities, and a wide range of specific comorbidities were less prevalent.

**Table 2:** Prevalence of comorbidities in the entire cohort and the three clusters

Comorbidity	Entire cohort (*n* = 211)	Cluster 1 (*n* = 99)	Cluster 2 (*n* = 65)	Cluster 3 (*n* = 47)
Total number of comorbidities (SD)	2.7 (2.1)	1.4 (1.3) **	4.2 (2.2) **	3.3 (1.4) *
Airway obstruction, *n* (%)	20 (9.5%)	11 (11.1%)	3 (4.6%)	6 (12.8%)
Obstructive sleep apnea, *n* (%)	19 (9.0%)	6 (6.1%)	9 (13.8%)	4 (8.5%)
Thromboembolic disorders, *n* (%)	10 (4.7%)	4 (4.0%)	2 (3.1%)	4 (8.5%)
Pulmonary hypertension, *n* (%)	20 (9.5%)	0 (0.0%) **	18 (27.7%) **	2 (4.3%)
Arterial hypertension, *n* (%)	117 (55.5%)	36 (36.4%) **	53 (81.5%) **	28 (59.6%)
Ischemic heart disease, *n* (%)	37 (17.5%)	1 (1.0%) **	35 (53.8%) **	1 (2.1%) **
Atrial fibrillation, *n* (%)	18 (8.5%)	5 (5.1%)	12 (18.5%) **	1 (2.1%)
Other arrythmias, *n* (%)	5 (2.4%)	1 (1.0%)	3 (4.6%)	1 (2.1%)
Heart failure, *n* (%)	25 (11.9%)	3 (3.0%) **	21 (32.3%) **	1 (2.1%) *
Heart valve disease, *n* (%)	8 (3.8%)	2 (2.0%)	6 (9.2%) *	0 (0.0%)
Cerebrovascular disease, *n* (%)	19 (9.0%)	4 (4.0%) *	12 (18.5%) **	3 (6.4%)
Peripheral artery disease, *n* (%)	8 (3.8%)	1 (1.0%)	4 (6.2%)	3 (6.4%)
Vascular disease, *n* (%)	2 (1.0%)	0 (0.0%)	2 (3.1%)	0 (0.0%)
Renal insufficiency, *n* (%)	7 (3.3%)	2 (2.0%)	5 (7.7%) *	0 (0.0%)
Gastro-esophageal reflux disease, *n* (%)	50 (23.7%)	26 (26.3%)	10 (15.4%)	14 (29.8%)
Liver disease, *n* (%)	9 (4.3%)	4 (4.0%)	4 (6.2%)	1 (2.1%)
Hypothyroidism, *n* (%)	28 (13.3%)	0 (0.0%) **	5 (7.7%)	23 (48.9%) **
Osteoporosis, *n* (%)	26 (12.3%)	0 (0.0%) **	4 (6.2%)	22 (46.8%) **
Diabetes mellitus, *n* (%)	43 (20.4%)	3 (3.0%) **	34 (52.3%) **	6 (12.8%)
Anemia, *n* (%)	8 (3.8%)	3 (3.0%)	5 (7.7%)	0 (0.0%)
Obesity, *n* (%)	69 (34.5%)	22 (24.2%) **	27 (42.2%)	20 (44.4%)
Lung cancer, *n* (%)	0 (%)	0 (%)	0 (%)	0 (%)
Non-lung cancers, *n* (%)	24 (11.4%)	9 (9.1%)	9 (13.8%)	6 (12.8%)
Depression, *n* (%)	18 (8.5%)	3 (3.0%) **	2 (3.1%)	13 (27.7%) **
Anxiety, *n* (%)	3 (1.4%)	0 (0.0%)	1 (1.5%)	2 (4.3%)

Values are presented as *n* (%) or mean with standard deviation (SD) [11]. Results from one cluster differing significantly from the two other clusters in combination are marked with: * p < 0.05; ** p < 0.01

The first map shows the location of the three clusters. The location of each patient on the map and the clusters borders (marked with black lines) are constant. The presence or absence of an individual comorbidity (one map per comorbidity) for patients in a given part of the map is indicated by a fitted color scale (Red: high frequency; Green: moderate frequency; Blue: low frequency). *GERD*: gastro-esophageal reflux disease; *C1*: cluster 1; *C2*: cluster 2; *C3*: cluster 3.

In cluster 2, patients were older with a majority of males, a larger proportion on LTOT, and more often with higher ILD-GAP scores (i.e., more severe disease besides male gender and older age). The total number of comorbidities was highest in this clusters, and patients suffered more frequently from cardiovascular diseases, diabetes, and renal insufficiency.

Patients in the third cluster were predominantly women with shorter 6MWD. They had more comorbidities than the two other clusters in combination. Cardiac diseases were less prevalent, whereas hypothyroidism, osteoporosis and depression were more frequent.

### Longitudinal analyses

**Fig. 2:**
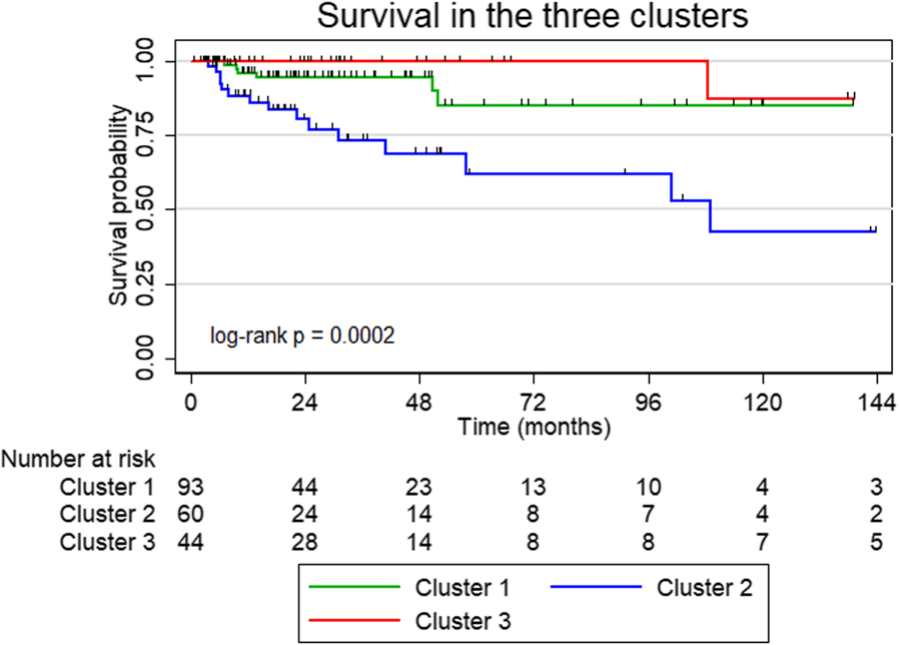
Survival in the three clusters

Mortality and changes in pulmonary function are presented in Table 3 and Fig. 2. The best survival was observed in cluster 3, whereas patients in cluster 2 had the worst prognosis, also after adjustment for GAP-ILD index and pack years. No significant difference in cause of death was observed in the three clusters (p = 0.17), potentially because of the low numbers.

**Table 3:** Mortality analyses and changes in pulmonary function during follow-up

Parameter	Cluster 1 (*n* = 99)	Cluster 2 (*n* = 65)	Cluster 3 (*n* = 47)
Cause of death, *n* (%)			
Respiratory	4 (66.7%)	4 (23.5%)	1 (33.3%)
Cardiovascular	0 (0.0%)	7 (41.2%)	0 (0.0%)
Unknown	2 (33.3%)	6 (35.3%)	2 (66.7%)
Mortality, univariate (95% CI)	1.26 (0.31 to 5.09)	5.58 (1.62 to 19.28)	Ref
Mortality, multivariate (95% CI)	2.19 (0.39 to 12.37)	5.83 (1.21 to 28.06)	Ref
ΔFVC, % predicted (95% CI)	− 1.9 (− 3.2 to − 0.6)	− 1.8 (− 3.0 to − 0.6)	− 1.6 (− 2.8 to − 0.3)
ΔDLCO, % predicted (95% CI)	− 0.7 (− 2.2 to 0.7)	− 1.8 (− 3.2 to − 0.4)	− 0.7 (− 2.3 to 0.9)
Δ6MWD, m (95% CI)	− 8.5 (− 22.7 to 5.7)	− 12.0 (− 20.1 to − 3.8)	− 10.9 (− 19.4 to − 2.4)

Data are presented as frequencies, hazard ratios (Cox regression analyses) or change pr. year (linear mixed effects models) with 95% confidence intervals. Median follow-up time was 1.8 years. Multivariate Cox regression analyses were adjusted for ILD-GAP index and pack years. Δ: Change per year; *FVC*: Forced vital capacity; *DLCO*: diffusion capacity of the lung for carbon monoxide; *6MWD*: 6-min walk test distance; *Ref.*: Reference group

A small decline in FVC% predicted per year was observed in all three clusters, but no difference between clusters was found (p = 0.94). In addition, patients in cluster 2 had a small decline in DLCO% predicted per year, but no difference in slopes between clusters could be proven (p = 0.49). Likewise, 6MWD declined in cluster 2 and 3, but the three slopes were not significantly different (p = 0.91).

Patients in cluster 1 had fewer respiratory hospitalizations than patients in the rest of the cohort (p = 0.021), whereas patients in cluster 2 had more admission days due to HP exacerbations compared to the other two clusters (p = 0.036).

## Discussion

In this study, we report the associations between comorbidities in patients with fHP using an unsupervised machine learning technique to identify clusters of comorbidities which could represent distinct phenotypes of fHP with diverging prognoses. We identified three new clusters of patients with fHP based on specific comorbidity profiles and found a higher mortality and more respiratory hospitalizations among patients in cluster 2, but no difference between the three clusters in pulmonary function or exercise capacity trajectories was shown.

The worse prognosis for patients in cluster 2 could be related to their comorbidity profile with more cardiovascular diseases. The cluster was dominated by older males and their survival was worse even after adjustment for the higher ILD-GAP index and pack years. Also, the increased rate of long-term oxygen therapy in this cluster possibly indicates disease progression. They might also have a more fibrotic pathology compared to patients in cluster 1, who had the highest lymphocyte count in BAL, which could indicate a more mixed inflammatory and fibrotic pathology [15, 16].

Cluster analysis is a well-established method which can be used to analyze associations between variables in complex data sets. In the field of ILD, cluster analyses have only been used in a limited number of studies, and we chose this novel approach to further explore the complex relationship between multiple comorbidities and their prognostic impact on patients with HP. The advantage of this unsupervised method is that no pre-defined associations are incorporated into the model allowing for more unbiased results. Furthermore, clusters of comorbidities could identify distinct phenotypes in fHP with different treatable traits. This approach brings focus on diagnosis and treatment of specific comorbidities in patients from different clusters, thus potentially improving prognosis and quality of life.

Cluster analyses including comorbidities or solely based on comorbidities have been reported in other ILD cohorts. Wong et al. identified two clusters in patients with fHP based on a combination of comorbidities, age, gender, and smoking pack-years, but primarily comorbidities and gender distinguished the clusters [17]. Comparable to our results, one cluster was dominated by males and the other by females with gastro-esophageal reflux disease and obstructive sleep apnea, and no difference in disease progression was seen. However, similar survival was seen in both clusters, whereas we found increased mortality in cluster 2. This difference could be caused by dissimilar cluster algorithms and registration of comorbidities as in the study mentioned, only comorbidities included in the Charlson comorbidity index were used, thus limiting the comorbidities available for cluster analyses. This approach excluded arterial and pulmonary hypertension, which are important prognostic factors in fHP [11, 18]. These comorbidities were more prevalent in cluster 2 in the present study and could to some extent explain the higher mortality in this cluster, thus emphasizing the importance of the choice of comorbidities for analysis based on the risk profile of a specific disease instead of a more generalized approach.

In patients with IPF, four clusters of comorbidities have been reported[8] and in unclassifiable ILD, three comorbidity clusters were identified [19]. Similar to our results, a cluster of patients with few comorbidities and a cluster dominated by cardiovascular diseases and male patients were found in both cohorts. This supports the robustness of these clusters. Emphysema was prevalent in the last clusters in IPF and unclassifiable ILD, but emphysema was not registered in the present study. The proportion of smokers and the number of pack years in IPF and unclassifiable ILD was much higher than in our cohort and thus, emphysema is probably less prevalent in patients with fHP as in the present study. On the other hand, a larger proportion of women with fHP compared to the two other types of ILD led to a separate comorbidity profile dominated by conditions more prevalent in women. In contrast to our findings, no difference in mortality was found in the two other studies, which could be explained by different follow-up times and mortality rates, as the impact of comorbidities on mortality has been shown in IPF [9, 20].

A strength of this study is the structured registration of comorbidities. Furthermore, all patients were diagnosed in a specialized ILD center suggesting a diagnosis with moderate to high confidence [21]. However, a limitation is the risk of missing or misclassification and underreporting of comorbidities in this retrospective study, which could influence the results of the study. Furthermore, treatment of HP and comorbidities was not accounted for in this study, and these interventions might affect both disease course and prognosis. Cluster analyses are well suited for investigation of relationships in large, complex data sets that would not otherwise have been evident. Still, such analyses are exploratory and should be confirmed in future studies.

## Conclusions

We identified three clusters with distinct comorbidities which could represent phenotypes in fHP not previously recognized. Mortality and respiratory hospitalizations were higher in the cluster dominated by cardiovascular diseases, but no differences in pulmonary function or exercise capacity trajectories were found. These clusters could reflect phenotypes in fHP with different treatable traits. This approach brings focus on diagnosis and treatment of specific comorbidities in patients from different clusters, thus potentially improving prognosis and quality of life.

## Data Availability

The datasets used and/or analyzed during the current study are available from the corresponding author on reasonable request.

## Abbreviations

6MWD: 6-Minute walk test distance
BAL: Bronchoalveolar lavage
DLCO: Diffusing capacity of the lung for carbon monoxide
FEV1: Forced expiratory volume in 1 s
fHP: Fibrotic hypersensitivity pneumonitis
FVC: Forced vital capacity
HP: Hypersensitivity pneumonitis
HRCT: High-resolution computed tomography
LTOT: Long term oxygen therapy
ILD: Interstitial lung diseases
ILD-GAP: Interstitial lung disease gender, age, and physiology
IPF: Idiopathic pulmonary fibrosis
IQR: Interquartile range
SD: Standard deviation
TLC: Total lung capacity
